# Any better? A follow-up content analysis of adolescent sexual and reproductive health inclusion in global financing facility country planning documents

**DOI:** 10.1080/16549716.2024.2315644

**Published:** 2024-06-19

**Authors:** Ulla Walmisley, Mary V. Kinney, Joël Arthur Kiendrébéogo, Yamba Kafando, Asha S. George

**Affiliations:** a School of Public Health, University of the Western Cape, Cape Town, South Africa; b Department of Public Health, University Joseph Ki-Zerbo, Ouagadougou, Burkina Faso; c Recherche pour la Santé et le Développement (RESADE), Ouagadougou, Burkina Faso; d Heidelberg Institute of Global Health, Medical Faculty and University Hospital, Heidelberg University, Heidelberg, Germany; e Institute of Tropical Medicine, Department of Public Health, Antwerp, Belgium

**Keywords:** Global Financing Facility for Women, Children and Adolescents: Examining National Priorities, Processes and Investments, Adolescent health, content analysis, development assistance, gender, global financing facility, health financing, multi-sectoral action, social determinants, world bank

## Abstract

**Background:**

The Global Financing Facility (GFF) supports national reproductive, maternal, newborn, child, adolescent health, and nutrition needs. Previous analysis examined how adolescent sexual and reproductive health was represented in GFF national planning documents for 11 GFF partner countries.

**Objectives:**

This paper furthers that analysis for 16 GFF partner countries as part of a Special Series.

**Methods:**

Content analysis was conducted on publicly available GFF planning documents for Afghanistan, Burkina Faso, Cambodia, CAR, Côte d’Ivoire, Guinea, Haiti, Indonesia, Madagascar, Malawi, Mali, Rwanda, Senegal, Sierra Leone, Tajikistan, Vietnam. Analysis considered adolescent health content (mindset), indicators (measure) and funding (money) relative to adolescent sexual and reproductive health needs, using a tracer indicator.

**Results:**

Countries with higher rates of adolescent pregnancy had more content relating to adolescent reproductive health, with exceptions in fragile contexts. Investment cases had more adolescent content than project appraisal documents. Content gradually weakened from mindset to measures to money. Related conditions, such as fistula, abortion, and mental health, were insufficiently addressed. Documents from Burkina Faso and Malawi demonstrated it is possible to include adolescent programming even within a context of shifting or selective priorities.

**Conclusion:**

Tracing prioritisation and translation of commitments into plans provides a foundation for discussing global funding for adolescents. We highlight positive aspects of programming and areas for strengthening and suggest broadening the perspective of adolescent health beyond the reproductive health to encompass issues, such as mental health. This paper forms part of a growing body of accountability literature, supporting advocacy work for adolescent programming and funding.

## Paper context

- **Main findings:** Adolescent health content is inconsistently included in the Global Financing Facility country documents, and despite strong or positive examples, the content is stronger in investment cases than project appraisal documents, and diminishes when comparing content, indicators and financing.
- **Added knowledge:** Although adolescent health content is generally strongest in countries with the highest proportion of births before age 18, there are exceptions in fragile contexts and gaps in addressing important issues related to adolescent health.
- **Global health impact for policy and action:** Adolescent health programming supported by the Global Financing Facility should build on examples of strong country plans, be more consistent in addressing adolescent health, and be accompanied by public transparency to facilitate accountability work such as this.

## Introduction

Adolescence – the life stage between 10 and 19 years of age – is a critical juncture for physical, cognitive, emotional, and social development. It presents a window of opportunity for health promotion which delivers a triple dividend of benefits across generations: for adolescents themselves, in their adult lives, and for their children [1,2]. WHO notes that ‘for every dollar invested in adolescent health, there is an estimated ten-fold health, social and economic return’ [1, p. 3]. Adolescents are recognised as key to achieving the Sustainable Development Goals [3], and the United Nations has called for expanding investment in child and adolescent health, to encompass actions which allow adolescents to ‘thrive’ rather than simply survive. The Global Financing Facility (GFF), a multi-donor trust fund hosted by the World Bank, stated adolescent health to be one of its priorities [4,5]. The GFF operates as a platform to catalyse collective action and resources for countries with the greatest reproductive, maternal, newborn, child, adolescent health, and nutrition (RMNCAH-N).

A small but growing body of work has begun to independently examine the work of the GFF, including from an operational perspective [6–9], exploring its impact or potential impact [6,10] and examining transparency [11]. Comparative analysis of global health initiatives, including GFF, [FGH study] has led to recognition of the importance of further learning about policy and financing effects of donor assistance [12]. George et al. [13] examined how commitments to adolescent health made by the GFF at global level translated into plans for the first 11 GFF partner countries through a content analysis of GFF country-level planning documents: investment cases (ICs) and project appraisal documents (PADs). George et al. [13] found examples of adolescent health inclusion, especially relating to teenage pregnancy. However, discrepancies were evident between countries. Additionally, adolescent health content faded as documents went from describing programming priorities to identifying indicators and committing funds. The paper concluded that, while the GFF, partner donors and countries must more consistently support the service delivery elements of adolescent health, funding should also be directed to address the main social and systems drivers of adolescent health, including gender and multi-sectoral efforts.

Subsequent to the above analysis, the GFF 2021–2025 Strategy [14] was launched. Reflecting global action on gender inequality [15], its second strategic directions emphasised equity and adolescent health, and reflected priorities in the GFF Roadmap for Advancing Gender Equality [16]. In 2022, the GFF published guidance for leveraging results-based financing to strengthen adolescent health outcomes [5], noting the insufficient progress made in this area. Against this backdrop and building on George et al. (2021), this paper examines adolescent-focused content in 16 additional GFF partner countries using the same method of analysis which examines programming content (mindset), indicators (measure) and investment (money). The paper also adds further understanding by categorising countries by adolescent health needs and reviewing commitments accordingly. This paper forms part of a Special Series aimed at understanding policy content and processes related to the GFF in recipient countries in an effort to promote accountability of and learning about this global health initiative and its role in galvanising action for those most left behind [17].

## Methods

This is a descriptive study that analyses the policy content of GFF country level planning documents (ICs and PADs). The investment case (IC) is meant to articulate national RMNCAH-N priority areas for funding [8], with an aim to mobilise funding from national governments, the World Bank, and other donors, including the private sector. The GFF grant acts as an incentive for governments to prioritise RMNCAH-N in the credit or loans provided by the World Bank and signed for in Project Appraisal Documents (PADs) [8]. As per the previous work done by George et al. [13], this study applied the same data collection tools and method of analysis and advances it by noting and describing how these aspects of planning documents are linked to country contexts.

In undertaking the policy content analysis, we followed the 4-phase READ approach for document analysis in health policy research [18], with steps consisting of (1) readying our materials, (2) extracting data, (3) analysing data and (4) distilling findings.

### Step 1: Readying materials

We include all GFF countries that were not assessed in the first paper, had at least one planning document available on the GFF website or that were provided by the Secretariat by October 2021. The 16 countries included are Afghanistan, Burkina Faso, Cambodia, CAR, Côte d’Ivoire, Guinea, Haiti, Indonesia, Madagascar, Malawi, Mali, Rwanda, Senegal, Sierra Leone, Tajikistan, Vietnam. Feedback from the GFF in 2023 revealed that the IC for Guinea, which appeared on the website, was an outdated version, and that Côte d’Ivoire’s monitoring and evaluation framework had not been posted at the time of data collection. We therefore expanded the data set to include these two more recent documents. Afghanistan, Tajikistan, and Vietnam did not have ICs available, while Madagascar and Sierra Leone did not have PADs. The date of the publication is not included on all of the documents; only seven countries have publication dates for both document. Of these, two had PADs that pre-dated the IC (Burkina Faso and Central African Republic).

### Step 2: Extracting data

Data were extracted using the same template and methods applied in the prior analysis of adolescent health (Supplementary file 1) [13]. Specific search terms were applied, and information gathered using a template to assess the placement and emphasis of adolescent-specific content in documents. The data extraction tool includes three interrelated framings or lenses: 1) a service delivery lens focussing on health conditions and interventions; 2) a societal lens highlighting the social determinants of health, namely gender; and 3) a systems lens which examines the engagement of actors inside and outside the health system.

Four authors were involved in the data extraction process (JAK, YK, MK, UW). The team reviewed the data extraction tools collectively and conducted parallel data extraction on one country to ensure consistency of coding and approach before extracting data on assigned documents. This team met on a regular basis to discuss the extraction process and align efforts.

To help contextualize our analysis, we extracted data on demographic context related to adolescent health for each country from existing databases [19,20]. Any other country context information apart from these selected indicators were drawn from the assessed GFF country planning documents.

### Step 3 & 4: Analysing and distilling data

**Figure 1.**
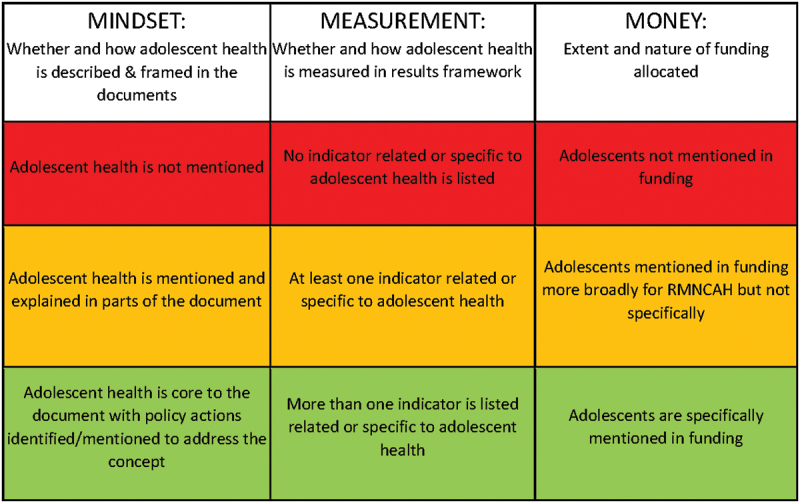
Mindset, measurement, money (M^3^) grading for inclusion of adolescent health in GFF planning documents.

Content analysis is used as a systematic process for reviewing the documents [21]. We noted whether there is alignment between priorities in ICs and PADs, and chronology of ICs and PADs. After re-reading the completed extracts, a summary document was developed for each country to further synthesize the results. Then, using these summaries, we applied a scoring system to grade the extent of adolescent inclusion across three ‘components’: adolescent inclusion in document content (*mindset*), indicators (*measurement*) and funding (*money*) (Figure 1) [17]. This approach permits comparison within and between documents as well as comparison between countries.

Additionally, we selected one indicator as a marker of a countries’ adolescent health needs: the proportion of births to women younger than age 18% births under – 18). We chose this statistic because it is a standard indicator drawn from the 2021 State of the World’s Children Report [19] and is reflective of individual, economic, sociocultural and health service-related factors for adolescent health [22]. Using this statistic, countries were placed into three groups based on the magnitude of this indicator. Group 1 countries had <10% births under-18, Group 2 countries had 10–25% births under-18, and Group 3 countries had >25% births under-18.

Analysis workshops in September 2021 and October 2022 enabled further synthesis of the results, alignment in approach, and discussion of results. Presentations were made to the Countdown to 2030 regional meeting in December 2022 and to the GFF Secretariat in March 2023 for feedback.

### Ethics

No ethical or special permissions were required for this study as it did not involve research on human subjects, and source documents are publicly available.

## Results

We start with contextualising the extent and nature of adolescent health needs by reviewing the demographic and health characteristics of adolescents in the countries in focus. We then consider the extent to which adolescents were included across the M^3^ components (mindset, measure, money) for each country’s IC and PAD, with each country grouped by extent of births under age 18. Finally, we focus on countries with the highest number of births under age 18 and examine the emphasis given to adolescents through three analytic lenses (service delivery, societal, systems).

### Demographic and health contexts of adolescents in countries

Adolescents made up approximately a fifth to a quarter of the population in all countries under examination, ranging from 17% in Indonesia to 26% in Central African Republic (Table 1). There was much wider variation, however, in health and social indicators for adolescents across these countries.

**Table 1.** Adolescent health and demographic indicators, ordered by national under-18 birth rate.

Country Groups: (% births by women <18 yrs.)	% Births by age 18 2015–2020	Countries	% Demand for FP satisfied by modern methods: 15–19 yrs.	Child marriage: % Married by 15 years	% Secondary school completion: Females	% Secondary school completion: Males	% Population that are adolescent: 10–19 yrs.
Group 1 [<10%]	1	Tajikistan	18	0	63	80	19
	5	Vietnam	60	1	61	50	14
	6	Rwanda	84	0	16	19	22
	7	Indonesia	82	2	37	40	17
	7	Cambodia	46	2	20	20	19
Group 2 [10–25%]	14	Haiti	31	2	16	17	21
	16	Senegal	25	9	10	11	23
	20	Afghanistan	21	4	14	32	25
	25	Cote d Ívoire	18	7	15	17	23
Group 3 [>25%]	28	Burkina Faso	51	10	2	6	24
	31	Malawi	62	9	13	15	25
	31	Sierra Leone	32	13	18	27	23
	36	Madagascar	51	13	15	16	23
	37	Mali	31	16	12	23	25
	39	Guinea	33	17	13	22	24
	43	CAR	14	26	6	8	26

Data source: United Nations Children’s Fund Global databases https://data.unicef.org/resources/sowc-2021-dashboard-and-tables/ (Accessed 28 August 2023).

The indicator ‘% births under-18’ varied widely between countries, from 1% in Tajikistan to 43% in Central African Republic. The percentage of demand for family planning satisfied by modern methods also ranged from 14% in Central African Republic to 84% in Rwanda, and girls married by 15 years of age ranged from 0% in Tajikistan and Rwanda to 26% in Central African Republic. Level of demand satisfaction for modern family planning did not appear to be consistently reflected in under-18 birth rates, exemplified by Tajikistan where 18% of demand for modern family planning was met but the under-18 birth rate was 1%.

Secondary school completion for girls ranged from 2% in Burkina Faso to 63% in Tajikistan. For boys, it ranged from 6% in Burkina Faso to 80% in Tajikistan. Only in Vietnam was there a higher rate of secondary school completion for girls than boys (64% for girls vs 50% for boys).

### How are adolescents included in the GFF planning documents?

**Figure 2.**
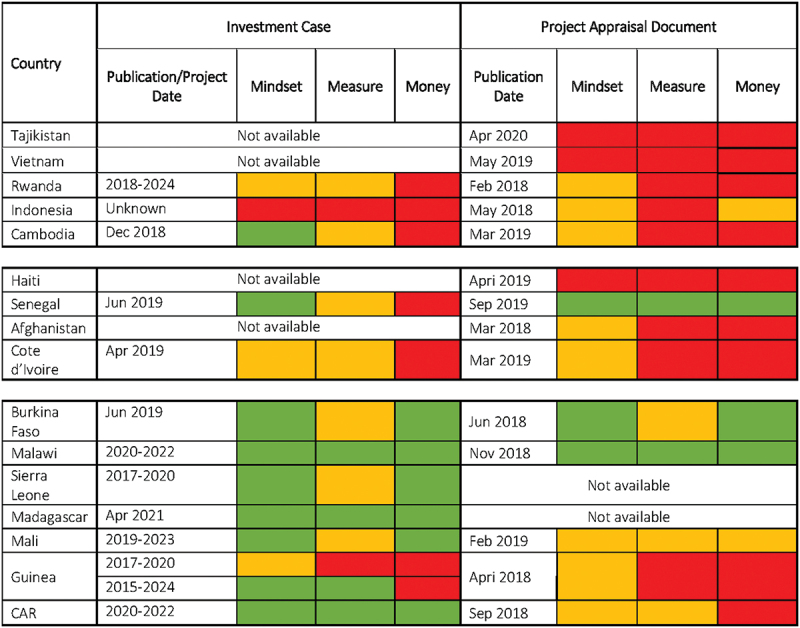
Extent to which adolescent health is included in Country ICs and PADs.Supplementary file 3: Summary analysis for Figure 2

Inclusion of adolescent health in the mindset, measures and money appeared broadly linked to % births under-18. This was observed for the 16 countries in focus here (Figure 2), and for the consolidated 27 countries, when including the previous analysis (Supplementary file 2). A summary of the analysis which informed Figure 2 is to be found in Supplementary file 3.

#### Group 1: Lower burden countries: Tajikistan, Vietnam, Rwanda, Indonesia, and Cambodia

In lower burden countries, where 10% or less of births were by women under age 18, mindset, measurement, and money components were mostly graded red, with some orange and only one green in Cambodia’s IC (discussed below). Overall, this indicated that content explicitly linked to adolescents was included marginally or not at all in these ICs and PADs. Inclusion of adolescent-specific content was expected to be low as the burden of adolescent pregnancy was also low relative to other countries. These country planning documents described contexts where poverty was declining, and the most pressing health needs related to childhood malnutrition and suboptimal human capital development. Country planning documents for Tajikistan, Rwanda, and Indonesia focussed on nutrition and health for early childhood, and Vietnam focussed on improving health outcomes at commune level.

Cambodia’s IC mindset included adolescents within the RMNCAH-N acronym, and noted that while fertility has declined generally, demand for adolescent reproductive health services was expected to increase with nearly a third of the population forecast to be women of reproductive age by 2020 [23]. The IC described the problem of teenage pregnancy and aimed to promote accessible and adolescent-friendly sexual and reproductive health services and the importance of providing sexual and reproductive health (SRH) education and parental education to adolescents. However, the PAD only briefly mentioned adolescents with no related indicators or allocated funding.

#### Group 2: Moderate burden countries: Haiti, Senegal, Afghanistan, and Côte d’Ivoire

In moderate burden countries, where 10–25% of birth were to women under age 18, adolescent health was still either marginally considered or mostly not at all (some orange, mostly red) with the exception of Senegal (Text Box 1). Other countries in this group were humanitarian or post-conflict settings that rarely mention adolescent health in their GFF document despite having a moderate burden (Figure 2). Haiti’s PAD did not mention them at all, and Afghanistan’s PAD mentions adolescents once in relation to widespread gender-based violence. Côte d’Ivoire’s was given an orange grade for mindset since the IC and PAD mentions key background elements related to adolescents including that half the population was under age 20; adolescents accounted for 14.8% of maternal deaths; and HIV was the leading cause of adolescent mortality. However, no further attention was paid to adolescents in terms of the measurement or money components.

Senegal was the only country in Group 2 that includes adolescents in GFF country planning documents (Text Box 1). The documents described a context of stability and economic growth but noted numerous challenges to adolescent health. Although the IC failed to address adolescents in terms of measurement or money, the PAD addressed adolescent health consistently across all three mindset, measurement, and money components.

**Text box 1:** Inclusion of adolescent health in Senegal’s documents.

1) *Mindset*: There is a high degree of consistency in the extent to which adolescent health is mentioned in the content of both the PAD and IC. Adolescent health falls under RMNCAH-H, and AYRH (Adolescent and Youth Reproductive Health). Senegal’s PAD describes its country context as politically stable, with a favourable economic outlook although youth experience higher levels of unemployment than other demographic groups, as well as educational and health care inequities. Early pregnancies and marriages are major concerns, particularly for rural adolescent girls from poor households, and with a low level of education. Both documents contain separate sections and project components dedicated to adolescent health, and adolescent rights are included. Annex 3 of the PAD is entitled ‘Special Focus on Adolescent Health in Senegal’, and describes the importance of investing in this demographic, the challenges it faces (mostly linked to SRH) and the need for empowering girls by reducing the rate of fertility and child marriage and improving educational attainment. Boys are discussed due to their key role in female empowerment. Cash transfers are proposed as a measure to incentivise girls’ continued education and vocational training, with the intention that empowerment and employment opportunities will reduce adolescent vulnerability and early pregnancy.
2) *Measurement*: While the IC does not include adolescent-specific indicators, the PAD Indicators are linked to outcomes which quantify benefits of the pilot cash transfer system for adolescents: Utilisation rate of modern contraceptive methods by adolescent girls in a relationship, aged 15–19; number of adolescent girls who benefited from cash transfers; adolescent girls’ pregnancy rate among the beneficiaries of cash transfer initiatives.
3) *Money*: The IC does not contain adolescent-specific financing, unlike the PAD. The PAD states that USD25 million of a total project budget of USD150 million is allocated to a project component promoting adolescent health and women’s empowerment.

##### Group 3 countries: Burkina Faso, Sierra Leone, Mali, Guinea, Madagascar, Malawi, Central African Republic

In high burden countries, where % under-18 births ranged from 28% in Burkina Faso to 43% in CAR, there was more attention to adolescent health, albeit not always consistently. Burkina Faso and Malawi were the only countries where priorities for adolescent health flagged in the IC correspond with commitments in the PAD and have the only PADs with strong adolescent-focussed content in the group. Adolescent health was core in the ICs for Mali and Central African Republic, supported by indicators and specified funding; but this was not followed through in their PADs. Guinea mentioned adolescents in relation to its high rate of teen pregnancy and early marriage, but although some strategies were included no indicators or financing were attached. Its IC stated that adolescent health would not benefit from a specific service package or budget, equating adolescent health needs with those of other targets (mother, child, newborn).

Since adolescent-specific inclusion was largely found in Group 3 countries, where needs were also highest, we describe content from this group in more depth. First, we describe how adolescents were framed and defined. We then described results by the three lens or frames applied by George et al. [13] including service delivery (health conditions addressed and programmes described), societal (i.e. gender considerations, inclusion of boys, social determinants of health) and systems (extent of multi-sectoral action, community engagement and inclusion of adolescent perspectives).

#### Emphasis and framing overall

In Group 3 countries, adolescents received consistent emphasis in available ICs, as well as in the PADs for Burkina Faso and Malawi, appearing throughout in executive summaries, introductions, situation analyses, budgets, and monitoring and evaluation sections, usually with specific subsections or project components dedicated to adolescent health. Adolescents did not receive consistent attention in the PADs of Mali, CAR, and Guinea.

When included, adolescents were discussed as a group vulnerable to health deficits (such as malnutrition, high fertility rates, low median age of first birth) and adverse social determinants (such as low rates of educational attainment, particularly for girls, and high youth unemployment). The potential for harnessing a positive demographic dividend was noted in Burkina Faso’s PAD and both of Mali’s documents but was not mentioned elsewhere. Adolescent rights were not mentioned, and nor was adolescence discussed as a critical developmental phase.

There was no formal definition of adolescence in any document, and some variation in age categorisation prevails across the documents. Most countries used the age range 15–19 to refer to adolescents, and there was some disparity where countries refer to younger adolescence, with the lower age limit varying between 9, 10 or 11 years of age.

##### Service delivery lens

Nutrition was linked to adolescents only in certain ICs (Burkina Faso, Sierra Leone, CAR). In contrast to nutrition, adolescent fertility, early pregnancy, and plans to improve uptake of family planning were widely described (Burkina Faso, Sierra Leone, Mali, Madagascar, Guinea). Adolescent-related gender-based violence and associated interventions were included in the IC’s of numerous countries (Burkina Faso, Sierra Leone, Mali, and CAR), but the PADs either did not address the issue or discuss it without particular reference to adolescents. HIV as a threat to adolescent health, and the need to increase coverage of adolescent HIV testing was mentioned within IC’s (Sierra Leone, Mali and Madagascar, CAR) but omitted in corresponding PADs. Female genital mutilation was a commonly identified issued within IC’s (Burkina Faso, Sierra Leone, Madagascar, and CAR), and was included in both of Mali’s documents. Obstetric fistula was only specifically mentioned for adolescents in Mali’s IC. Abortion, as a consequence of early sexual debut, unsafe sex, and teen pregnancy was discussed in IC’s but not PADs (Mali, Sierra Leone, Burkina Faso, and CAR). Provision of safe abortion for adolescents was not mentioned by any country document, although Malawi’s IC allocated funding to assess the feasibility of legalising abortions and develop a workplan. This was not carried through in the PAD. CAR’s IC included post abortion care for adolescents as an indicator.

Provision of adolescent-friendly services were discussed in the IC’s of Burkina Faso, Sierra Leone, and Madagascar. Burkina Faso and Madagascar outline plans for integrated service packages, adapted to the needs of adolescents and youth, while Sierra Leone mentions provision of adolescent safe spaces. Corresponding PADs did not include this.

Schools, as avenues for strengthening adolescent health, were mentioned by several country ICs to varying degrees (Burkina Faso, Sierra Leone, Mali, and CAR). This ranged from acknowledgement of the need to retain youth in schooling, without specific related plans (Mali), to descriptions of school health programmes to promote SRH education and monitor health conditions (Burkina Faso and CAR), to detailed plans linking stipends and performance-based financing (PBF) to improved school enrolment and student retention, and use of schools as sites for health promotion (Sierra Leone).

The above plans generally did not specify budget allocations for particular adolescent programming items but allocated a percentage of the total budget to adolescent health. The use of PBF was an exception, which linked funding mechanisms directly to activities.

##### Societal lens

With regards to social determinants, all documents in group 3 countries (with exception of CAR’s PAD) stated that early, child or forced marriage negatively impacts maternal and child health linked to early sexual debut and high adolescent fertility, low socioeconomic status of women and girls, in conjunction with low educational attainment, and adverse health impacts. Only Guinea did not discuss early marriage as a driver of adolescent health.

Gender inequity as a social determinant of adolescent health was specifically discussed in the IC’s of Burkina Faso, Mali, and Madagascar, which variously noted that boys have higher levels of educational attainment (both of Burkina Faso’s documents), and that inequality underpins negative practices such as early marriage (Mali’s IC) and earlier female sexual debut (Madagascar and CAR’s IC’s).

Boys were briefly mentioned in these documents with the exception of Guinea and CAR’s PAD. For example, reference was made to engaging men and boys in community outreach to improve adolescent safety (Burkina Faso’s PAD), the need to also focus on boy’s education and upliftment (Sierra Leone’s IC) and in relation to determinants of condom use (Mali’s IC).

##### Systems lens

With regards to engaging with actors across health system levels, meaningful input of adolescents in planning was not mentioned within any planning document, besides Sierra Leone’s IC which states that social and behaviour change communication would be conducted using adolescent peer groups, and an annexure to Malawi’s IC which provided details of youth participation in planning.

Multisectoral engagement to support adolescent health by linking with sectors such as education were mentioned in most country documents (except for the PADs of CAR and Mali). Madagascar’s IC mentioned the Ministry of Youth and Sport, which fosters the legal, policy and strategic environment for youth access to reproductive health services. Burkina Faso (in its IC and PAD), as well as the ICs of Sierra Leone, Mali, Madagascar, and CAR, described community engagement and the involvement of CHW’s to increase access to, and use of, RMNCAH services specifically to improve adolescent health. Burkina Faso’s PAD mentioned that community health workers would expand civil registration to combat early and forced marriage, and Mali’s IC discussed strengthening mass communication by implementing a communication campaign on adolescent and youth friendly reproductive health through the media with the involvement of religious leaders and traditional communicators and other opinion leaders and raising awareness in schools and in the community. Guinea’s new IC stated that to ensure that commitments were properly monitored, and to promote the accountability of key actors in the fight against preventable maternal, newborn, child and adolescent deaths, civil society organizations would set up a dashboard.

Funding for particular multisectoral action was rarely described as these elements were collaborative and overlap with existing programming. For example, Sierra Leone described how the RMNCAH strategy would support scale up of an existing multisectoral model for adolescent health that was being rolled out between the Ministry of Education in collaboration with development partners, but did not allocate particular budget items to this point.

## Discussion

Overall, these new findings align with the previous study results whereby adolescents are mentioned to some extent in most country documents but are inconsistently featured across the ICs and PADs, fading in focus from mindset to measures and money. Critically, we found that adolescents are generally included in planning documents in countries where their needs are greatest, albeit not consistently. What is noteworthy is that Burkina Faso and Malawi both had slightly different foci in ICs than PADs, but both retained adolescent content – showing it is possible to meet commitments to adolescent programming even within a context of shifting or selective priorities.

The focus on adolescent health in countries with the highest burden of adolescent pregnancy is commendable, but we also found a lack of adolescent content in ICs from countries that are fragile, or with humanitarian settings. Given adolescent vulnerability in unstable settings [24], it is concerning that these countries made little or no mention of them in planning documents. While these countries may be resource constrained, efforts should include measures to protect and support their adolescent populations in addition to other vulnerable groups [24–26]. For these specific humanitarian settings and otherwise, regional bodies are key to engage with to align, leverage and sustain policy commitments.

The previous paper found that adolescent content in ICs was often not carried through to PADs [13], and we found the same – only three of the included PADs had strong adolescent emphasis (Senegal, Burkina Faso, and Malawi). This echoes other work on this topic, which found variable alignment between IC and PAD indicator use [6]. Keller et al. [5,p.22] note that ‘*differences might reflect a form of priority-setting aimed at establishing more conservative or realistic targets*’. While priority setting may influence which aspects of ICs appear in PADs, it is also important to recognize that PADs are just one funding source for ICs and that IC priorities may be funded by PADs that are not publicly available, or by other donors (May 2023 email correspondence between the GFF Secretariat and authors). The fact that adolescent-specific budget items appear less often in PADS, therefore, may not be an omission, but a consequence of being funded by other sources. Prioritisation of, and funding allocations from, donors and non-governmental organisations can be opaque [27], making it difficult to track how adolescent health is funded. For the purposes of external accountability research, it would be useful to encourage further transparency about financial allocations to enable a full understanding of what resources are directed to adolescent health programming.

Adolescents are a sizeable demographic within all countries, although health and wellbeing for adolescents vary widely and reflect diverse needs. Documents that include adolescent content generally have a strong focus on RMNCAH service delivery, teen pregnancy and family planning for adolescents [13]. Other related health conditions are less well addressed, such as fistula or mental health, despite these being consequences of adolescent pregnancy and contributing to multidimensional poverty and reduced educational outcomes [28,29]. The burden of adolescent mental health challenges in low- and middle income countries (LMIC), and associated socioeconomic costs indicates that advocacy and investment for strengthened psychosocial interventions is needed [29].

Appropriateness and sustainability of adolescent services may be strengthened by adolescent participation in planning [30,31]. Annexures in Malawi’s documents provide an example of a detailed description of adolescent participation in policy formulation at various stages, but overall, this is lacking. To optimise inclusion of adolescent perspectives, it would be helpful if adolescent participation is strengthened, and clearly reported, in future plans.

Rates of satisfaction in demand for modern contraceptives were not always linked to % births before age 18 (which is a retrospective figure) [32], exemplified by Tajikistan. This is possibly related to cultural and political factors. In the case of Tajikistan, access to contraceptives is limited by a requirement of parental consent for adolescents under the age of 18 to access family planning [33], while premarital sexual activity may be curtailed by the fact that Tajikistan is a Muslim country, which has become more conservative around the roles of women [34].

Post-abortion care without specific mention of safe abortion services for adolescents was a notable inconsistency in the documents. An absence of public discourse on the topic of abortion may not reflect actual service provision [35]. Support for abortion may be undertaken in strategic ways given sensitivities of parliamentary oversight over these financing agreements. However, this may challenge efforts to monitor progress externally.

Adolescent sexual and reproductive health services are supported by two main approaches – adolescent friendly health services and school health programmes [13]. Training for youth friendly services is critical in the success of adolescent and youth friendly service promotion [36], as adolescents have specific health needs, and providers should feel comfortable providing adolescent-specific care [37]. The health workforce has an important role in service acceptance to adolescents [36], and poor interpersonal skills and stigmatisation by providers are a recognised barrier to service use [38]. Extensive curricula are available for training in adolescent-friendly services in LMIC but are reportedly under-used in these contexts [37]. Country plans should include evidence-based programming for positive youth development [39].

Across countries, data reveal generally low rates of secondary school completion particularly for girls – revealing prevalent gender inequity [40]. The use of stipends has been found effective in reducing economic barriers to education – particularly crucial where the pandemic increased financial pressure on households [41]- but little evidence has been produced in LMIC, and further investigation is needed to advocate for the use of stipends in these contexts [42]. Senegal provides a good example in both documents of piloting stipends to encourage retention in schooling. This could be applied elsewhere, especially in light of multidimension benefits of education for health promotion [43] reduced adolescent fertility rates [44], and potential for improving adolescent health outcomes [45]. Some ICs include plans for norm shifting, through mass media or social media communication, peer support or community action, in line with the GFF policy [5] and literature [46,47], but needs to be included in PAD project components and monitored if attitudes and practices are to change [48].

Since publication of our previous paper, priority indicators for assessing progress on adolescent health have been released by the GAMA advisory group [49]. We found that – of all country PADs – only Senegal and Malawi contained multiple specific indicators for measuring project components for adolescent health. Other PADs usually contained only one indicator focussing on adolescents, and indicators used varied between countries. Given that GFF ICs and PADs maybe limited to 10 key indicators overall, it is critical to clarify the reasoning behind the adolescent health indicator selected and underline efforts to support comparative learning.

Adolescent health is frequently underfunded compared to other age groups [1,50]. In our study, countries that included adolescent health in their description of project components did link to specific funding, sometimes describing pilots for financial incentivisation at varying levels of the health system. To maximise financing for this underserved demographic, decision-makers should also consider cross-sector co-financing and make these linkages more explicit in the GFF documentsfor example, between education and health sectors, to facilitate meaningful structural change [51], and strengthen the use of financing levers which target adolescent-responsive action [5].

### Strengths and limitations

Our work sheds light on adolescent health policy at national level and informs GFF programming by bringing additional focus to commitments, flagging successes, as well as areas for strengthening. We used documents that were publicly accessible on the GFF website or provided by the Secretariat, but this may not comprehensively depict adolescent programming supported by the GFF. Once approved, GFF support continues to evolve through annual reviews and restructuring agreements. In certain countries, adolescent health is addressed by other Work Bank initiatives and cross-referenced in the GFF planning documents, such as the Sehatmandi project in Afghanistan [6,14], or the Sahel Women’s Empowerment project [52]. As a result, the GFF planning documents may not give a full picture of adolescent health programming in a country, nor the World Bank’s contribution to the it. Given this potential incomplete knowledge it is a challenge to make recommendations about adolescent health programming in countries and what the World Bank, and other partners involved in GFF, should or could be doing. Additional research and mapping of activities for adolescent health within each country would be needed for this purpose. Although greater transparency and knowledge management could facilitate future tracking, such as listing related World Bank projects in the GFF country portals. GFF could also consider establishing a platform to clarify how adolescent health being addressed and funded by various agencies and organisations as part of the broader RMNCAH continuum.

Our data set is made up of documents that were written and published a number of years ago. Since then, the evidence base and recommendations around adolescent health have made significant strides, reflected in subsequent GFF literature [4,5,53].

Finally, we work outside the GFF, and thus do not have in-depth knowledge of all the nuances involved. Nonetheless, to ensure continuity and rigour in our analysis, two of the authors (AG, MK) were involved in the previous analysis. We used standard templates for data analysis, engaged in regular meetings and several analysis workshops. The study was not commissioned by the GFF secretariat; however, they were informed about the study and provided feedback on the results and interpretation for consideration. AG also serves on the GFF Results Advisory Group and therefore brokered feedback from the GFF for the study.

## Conclusion

In complex and diverse contexts, adolescent health has been integrated into GFF planning documents for countries that face the most substantial challenges related to sexual and reproductive health. Programmatic elements could receive stronger support using validated measures and be clearly linked to budgetary provisions. Accountability work, such as this, can be used to advocate for inclusion of adolescent programming and funding for countries where it needs to be strengthened.

## Supplementary Material

Supplementary files.docx

## Data Availability

The datasets used and/or analysed in this study are available from the corresponding author on reasonable request. This paper is part of the *Global Health Action* Special Series, “Global Financing Facility for Women, Children and Adolescents: Examining National Priorities, Processes and Investments”, available in Volume 17-01.
