# Supplementary material for: Any better? A follow-up content analysis of adolescent sexual and reproductive health inclusion in global financing facility country planning documents
Source: Glob Health Action. 2024 Jun 19;17(1):2315644. doi: 10.1080/16549716.2024.2315644 (PMC11188955; doi:10.1080/16549716.2024.2315644)
Supplement: Supplementary files.docx [file ZGHA_A_2315644_SM0617.docx]

# Supplementary file 1: Search terms and data extraction template

**GFF adolescent health analysis country data extraction template**

*Instructions:*

- *Save file as: Country name data extraction date [DDMMYYYY]*
- *Add information where there is instruction in yellow highlight*
- *Add in the data for each indictor from the excel of data*
- *Write summary statements to synthesize the information for each question*
- *Complete data extraction by recording summary points or copying in text from document in bullet form to respond to the below questions*
  - *Include page numbers for content*
  - *Include screen shots of relevant tables*
- *To find the information use the following search terms:*
  - *adolescent health: adolescent, teen, youth, young*
  - *gender: gender, men, male (female/ women should be found via men/male), girls, boys)*
  - *multisectoral: multisectoral, intersectoral with hyphen or not, school, education)*
  - *community: community, civil society, CBO*

Country: ADD COUNTRY NAME

Prepared by ADD YOUR NAME

Date: ADD DATE OF COMPLETION

**General**

Investment case proposal: Title, Date published, Total pages: xx

PAD: Title, Date published, Total pages: xx

*Key country context details*

- - - Key adolescent health indicators

| **Country** | **Country name** |
| --- | --- |
| **% of population that are adolescent (10-19)** |  |
| **Adolescent (10-19) mortality rate (per 100,000), male** |  |
| **Adolescent (10-19) mortality rate (per 100,000), female** |  |
| **HIV incidence per 1,000 uninfected population - 15-19 yrs - 2016** |  |
| **% of women aged 20–24 who gave birth before age 18 2011-2016** |  |
| **Demand for family planning satisfied with modern methods - 15-19 yrs** |  |
| **Family planning for adolescents without spousal or parental consent** |  |
| **Child marriage (%) Married by 15** |  |
| **Secondary completion rate, % (upper, females)** |  |
| **Secondary completion rate, % (upper, males)** |  |
| **Legal status of abortion** |  |

1. ***Placement of adolescent health***
   1. *Where is it in the document (ToC, Foreword, Situation Analysis, Indicators, Budget, etc)*
      - *Is it consistently mentioned throughout the document, or does attention narrow or disappear as you move through to operational details, budgets, indicators? How so or why not?*
      - *Any budget details at all?*
   2. *Is it mentioned mainly as part of a larger acronym/ as an ‘add on’ within other topics or are there independent sections/ detailed analyses and programmes specifically for adolescents?*
      - *If there are independent sections note what they are*
2. ***Definition and description of adolescents***
   1. *Is there a definition? If so, what is it?*
   2. *What are the age ranges listed? What are the age ranges linked to?*
   3. *Are boys mentioned as well as girls? How prominently? If so how?*
3. ***Framing,*** *are adolescents mainly listed as being*
   1. *health deficits to address*
   2. *instrumental for other health conditions, ie through linkages (eg adolescent SRHR has implications for premature childbirth)*
   3. *as particularly vulnerable (historical neglect)*
   4. *as opportunities:*
      - 1. *Economic dividend for investment (demographic dividend)*
        2. *More broader positive definition of health and well-being*
           1. *Is adolescence as a key developmental phase mentioned? If so how?*
           2. *Are adolescent rights listed? How?*
4. ***Service delivery lens (conditions and interventions)***
   1. *Are sexual and reproductive health issues and interventions mentioned, how?*
      - 1. *Family planning*
        2. *Safe abortion statistics and services*
        3. *HIV*
        4. *FGM/ fistula*
        5. *Adolescent friendly and responsive services*
        6. *School health programmes/sex education*
   2. *Other conditions & interventions mentioned e.g. mental health, nutrition, violence*
   3. *What is missing?*
   4. *What non health care services/ interventions mentioned, or not?*

*e.g. GBV, nutrition, school health? Life skills?*

1. ***Societal lens (problem definition and response/ intervention)***
   1. *Any mention of rights, legal frameworks?*
   2. *Are there any social determinants underpinning the status of adolescents mentioned?*
      - *Marriage*
      - *Out of school*
      - *Conflict*
   3. *Is gender inequality mentioned? Gender norms? With adolescents or more broadly*
   4. *Is empowerment mentioned? In what ways? To what extent? With adolescents or more broadly*
   5. *What other aspects of vulnerability and inequality are listed, even if not linked to adolescents?*
   6. *What is not there?*
2. ***Systems lens (actors and levels that play an interactive role, adaptive responses)***
   1. *How are systems of governance and leadership listed for adolescents? In general?*
      - *Accountability mechanisms (eg clinic committees, RBF, etc)*
      - *Levels of the health system engaged*
   2. *Do the documents discuss what is the involvement of adolescent in planning, implementation, M&E? How is their voice and participation supported?*
   3. *Are other family forms mentioned? How? (godmothers, godfathers, parents groups, mother-in-laws)*
   4. *How is community engagement for adolescent health listed and for what purpose?*
   5. *What is listed about community independent of adolescents?*
      - *If CHWs mentioned, how?*
   6. *Are civil societal organisations related to adolescent health mentioned? How?*
   7. *Is private sector mentioned? How?*
   8. *What sectors/ ministries are listed related to adolescent health? What further detail is mentioned? What other areas are listed for multi-sectoral action?*
   9. *What is listed about multi-sectoral action independent of adolescents?*
3. ***M&E***
   1. *What type of results/ indicators are used (mortality, morbidity, social determinants)*
   2. *Are there specific RBF indicators for adolescents?*
   3. *Does mention of adolescent health disappear when it comes to M&E*
   4. *Any other M&E relevant for adolescents?*
4. ***Any other relevant comments or issues***
   1. *Relationship between IC and PAD e.g. in terms of consistency*

# Supplementary file 2: Adolescent health content analysis across all first 27 countries.

| **Country Groups** | **% births by women <18 yrs.** | **Country** | **Investment Case** | | | | **Project Appraisal Document** | | | |  |
| --- | --- | --- | --- | --- | --- | --- | --- | --- | --- | --- | --- |
|  |  |  | **Publication/Project Date** | **Mindset** | **Measure** | **Money** | **Publication Date** | **Mindset** | **Measure** | **Money** |  |
|  |  |  |  |  |  |  |  |  |  |  |  |
| **Group 1 <10%** | **1** | **Tajikistan** | **Not available** | | | | Apr 2020 |  |  |  |  |
|  | **5** | **Vietnam** | **Not available** | | | | May 2019 |  |  |  |  |
|  | **6** | **Rwanda** | 2018-2024 |  |  |  | Feb 2018 |  |  |  |  |
|  | **7** | **Indonesia** | Unknown |  |  |  | May 2018 |  |  |  |  |
|  | **7** | **Cambodia** | Dec 2018 |  |  |  | Mar 2019 |  |  |  |  |
|  |  |  |  |  |  |  |  |  |  |  |  |
| **Group 2 10-25%** | **14** | **Haiti** | **Not available** | | | | Apr 2019 |  |  |  |  |
|  | **16** | **Senegal** | Jun 2019 |  |  |  | Sep 2019 |  |  |  |  |
|  | **20** | **Afghanistan** | **Not available** | | | | Mar 2018 |  |  |  |  |
|  | **20** | **Guatemala** | 2016 |  |  |  | March 2017 |  |  |  |  |
|  | **21** | **Ethiopia** | Oct 2015 |  |  |  | Apr 2017 |  |  |  |  |
|  | **22** | **Uganda** | April 2016 |  |  |  | July 2016 |  |  |  |  |
|  | **23** | **Kenya** | Jan 2016 |  |  |  | May 2016 |  |  |  |  |
|  | **24** | **Bangladesh** | Mar 2017 |  |  |  | July 2017 |  |  |  |  |
|  |  |  | **Not available** | | | | Nov 2017 |  |  |  |  |
|  | **25** | **DRC** | Oct 2017 |  |  |  | March 2016 |  |  |  |  |
|  |  |  |  |  |  |  | March 2017 |  |  |  |  |
|  | **25** | **Cote d’Ivoire** | Apr 2019 |  |  |  | Mar 2019 |  |  |  |  |
|  |  |  |  |  |  |  |  |  |  |  |  |
| **Group 3 >25%** | **28** | **Burkina Faso** | Jun 2019 |  |  |  | Jun 2018 |  |  |  |  |
|  | **28** | **Tanzania** | 2016-2020 |  |  |  | May 2015 |  |  |  |  |
|  | **28** | **Cameroon** | 2017-2020 |  |  |  | Apr 2016 |  |  |  |  |
|  | **28** | **Nigeria** | 2017-2030 |  |  |  | May 2016 |  |  |  |  |
|  | **30** | **Malawi** | 2020-2022 |  |  |  | Nov 2018 |  |  |  |  |
|  | **31** | **Sierra Leone** | 2017-2020 |  |  |  | Not available | | | |  |
|  | **34** | **Liberia** | 2016-2020 |  |  |  | Jan 2017 |  |  |  |  |
|  | **36** | **Madagascar** | Apr 2021 |  |  |  | Not available | | | |  |
|  | **37** | **Mali** | 2019-2023 |  |  |  | Feb 2019 |  |  |  |  |
|  | **39** | **Guinea** | 2017-2020 |  |  |  | Apr 2018 |  |  |  |  |
|  |  | **Mozambique** | Apr 2017 |  |  |  | Nov 2017 |  |  |  |  |
|  | **43** | **CAR** | 2020-2022 |  |  |  | Sep 2018 |  |  |  |  |

**Key: Countries were grouped by the indicator “% births before age 18” - Group 1 <10% , Group 2 10-25%, Group 3 >25%**

***Note* – Some ICs do not state a date of publication. Project duration is used as a reference date where a publication date is not available**

# Supplementary file 3: Summary analysis for Figure 2

| **1. TAJIKISTAN** | | |
| --- | --- | --- |
| **IC** | Not available | |
| **PAD Focus: ECD** | **CONTENT** | The PAD doesn’t deal with adolescent health, it is focussed on ECD. “Youth” are mentioned in relation to the absence of sustainable job opportunities which has proven to be a key push factor incentivizing individuals to join extremist groups. The term “adolescent” appears once, as part of the RMNCAH acronym. |
|  | **INDICATORS** | None specific to adolescents |
|  | **INVESTMENT** | None specific to adolescents |
| **2. VIETNAM** | | |
| **IC** | Not available | |
| **PAD Focus: improving the quality and utilization of commune-level health service** | **CONTENT** | The PAD does not focus on or mention specifically adolescents. This PAD is focused on improving the quality and utilization of commune-level health services, with explicit mention to reaching vulnerable and disadvantaged populations such as ethnic minorities and those living in poor, remote, and mountainous provinces. |
|  | **INDICATORS** | None specific to adolescent |
|  | **INVESTMENT** | None specific to adolescents |
| **3. RWANDA** | | |
| **IC Focus: ECD** | **CONTENT** | Adolescent health is mentioned in relation to reducing anaemia in adolescent girls so that they are in optimal health as they reach child-bearing age. Adolescents are framed with the RMNCAH acronym, but not in a separate section and without great detail |
|  | **INDICATORS** | Baseline and targets for teen pregnancy, drawn from the Health Sector Strategic Plan, are included. They are not actual targets of the ECD plan of the IC, however. |
|  | **INVESTMENT** | None specific to adolescents |
| **PAD Focus: Nutrition** | **CONTENT** | Adolescent girls are are very briefly mentioned with no separate sections, in relation to improved nutrition for their reproductive health years. |
|  | **INDICATORS** | None specific to adolescents |
|  | **INVESTMENT** | None specific to adolescents |
| **4. INDONESIA** | | |
| **IC Focus: Nutrition** | **CONTENT** | No mention |
|  | **INDICATORS** | No indicator related or specific to AH is listed |
|  | **INVESTMENT** | Adolescents are not mentioned in funding |
| **PAD Focus: Nutrition** | **CONTENT** | Adolescent Health is mentioned in PAD mostly in relation to the high levels of adolescent pregnancy rates but overall investments are broad aimed at helping most vulnerable populations |
|  | **INDICATORS** | No specific indicators for adolescent health (indicators are intermediate results linked to broader strategies) |
|  | **INVESTMENT** | No specific investments but recognition that DLI’s for behaviour change communication and community investments will cover activities that target early marriage and adolescent pregnancy as drivers to stunting. |
| **5. CAMBODIA** | | |
| **IC Focus: RMNCAH-N** | **CONTENT** | IC is in the form of a PowerPoint presentation, so the format differs from the norm. Adolescents are discussed throughout (Priority interventions, key outcomes, indicators) |
|  | **INDICATORS** | Teenage pregnancy rate 15-19 years |
|  | **INVESTMENT** | Adolescents are not mentioned in funding (PPT lacks budget details) |
| **PAD Focus: Nutrition** | **CONTENT** | Adolescents are included under enhanced nutrition for pregnant and lactating women, but not mentioned in any depth. No separate sections |
|  | **INDICATORS** | No indicator related or specific to AH is listed |
|  | **INVESTMENT** | Adolescent specific line items appear in the cost estimate, (i.e., provision of SRH education and parental education to adolescents; promotion of adolescent friendly SRH services) but amount is allocated. |
| **6. HAITI** | | |
| **IC** | Not available | |
| **PAD Focus: Strengthening PHC and surveillance** | **CONTENT** | Adolescents are mentioned only twice, in a description of the GFF overall. The PAD outlines a plan based on the successful previous cholera response to strengthen PHC and improve surveillance and disease control. |
|  | **INDICATORS** | No indicator related or specific to AH is listed |
|  | **INVESTMENT** | Adolescents are not mentioned in funding |
| **7. SENEGAL** | | |
| **IC Focus: Reduction of maternal, neonatal, under-five years old, adolescent and youth mortality** | **CONTENT** | Adolescent health is highlighted in all sections of the IC, generally coupled with maternal, neonatal, child, and youth health as well as in separate sections. AH is a key priority of the document |
|  | **INDICATORS** | Includes the fertility rate of adolescents aged 15-19, and the rate of use of SRH services by adolescents aged 15-19. |
|  | **INVESTMENT** | No specific investments |
| **PAD Focus: Maternal, child and adolescent health** | **CONTENT** | Adolescents are mentioned throughout the document with a special focus in the annex on factors relating to adolescent health, mostly related to adolescent pregnancy. |
|  | **INDICATORS** | Indicators related specifically to adolescent health are included under “Promote adolescent health and women’s empowerment”: Utilisation rate of modern contraceptive methods by adolescent girls in a relationship, aged 15-19; Number of adolescent girls who benefited from cash transfers; Adolescent girls pregnancy rate among the beneficiaries of cash transfer initiatives |
|  | **INVESTMENT** | The above indicators are DLI – total project investment US$25 million (all from IDA not GFF) for adolescent health and women’s empowerment |
| **8. AFGHANISTAN** | | |
| **IC Focus: n/a** | Not available | |
| **PAD Focus: Sehatmandi project** | **CONTENT** | It is mentioned three times. Once as a group affected by the prevalence of gender-based violence; twice noting GFF has opportunity to focus on adolescent health |
|  | **INDICATORS** | Adolescents are not included |
|  | **INVESTMENT** | Adolescents are not mentioned in funding |
| **9. COTE D’IVOIRE** | | |
| **IC Focus: RMNCAH** | **CONTENT** | Adolescent health and needs are addressed very little in the IC. There is no specific section on AH. AH is mentioned as part of RMNCAH considered generally |
|  | **INDICATORS** | *In the "results framework" section where it is mentioned that the adolescent fertility rate was 124 ‰ in 2016 according to the MICS survey.* |
|  | **INVESTMENT** | Adolescents are not mentioned in funding |
| **PAD Focus: Strategic purchasing and alignment resources** | **CONTENT** | AH is generally included under RMNCAH-N. Specific mention to adolescents include: i) support for and incorporate learning from SWEDD to identify the best channels to reach adolescents with contraceptive services; and ii) high teenage pregnancy rate accounting for 30% of pregnancies and 14.8% of maternal deaths. No separate sections |
|  | **INDICATORS** | Adolescents are not included |
|  | **INVESTMENT** | Adolescents are not mentioned in funding |
| **10. BURKINA FASO** | | |
| **IC Focus: RMNCAH-N, Civil registration & Vital statistics** | **CONTENT** | Adolescent health appears consistently in the various sections of the IC, including in the package of high impact interventions and priorities, and within separate sections |
|  | **INDICATORS** | The Performance Framework only has one indicator: “Contraceptive prevalence in the adolescent/youth population”. |
|  | **INVESTMENT** | There is a planned investment included for adolescent health of USD 143,104,309 over five years (2019 - 2023), representing 7,9% of the total budget of the IC. |
| **PAD Focus: Health Services Reinforcement** | **CONTENT** | Adolescent Health is consistently mentioned throughout the PAD and is included in M&E and budget sections. TA separate section is project component "Promoting family planning with a focus on adolescent health and well-being" |
|  | **INDICATORS** | The Results Framework only has one indicator: “Proportion of adolescent girls delivering in facilities in the strategic purchasing program receiving postpartum family planning” |
|  | **INVESTMENT** | There is a budget mentioned for “Subcomponent 2.1: Strengthening maternal, newborn, child, and adolescent health service delivery (US$5 million IDA, US$8 million GFF), which comprises of 3 subcomponents, the second one being: “Sub-component 2.1.2: Promoting family planning with a focus on adolescent health and well-being” |
| **11. MALAWI** | | |
| **IC Focus: RMNCAH-N** | **CONTENT** | Adolescents are discussed throughout the document and are specifically discussed in a section “Reproductive and Adolescent Health”, and multisectoral action between ministries of health and education are detailed. Adolescent participation is noted, with stakeholders in the budget workshop listed as a youth champion and the National Youth Council of Malawi. The youth champion also participated in the M&E workshop. They are included in the budget and indicators. Boys are not mentioned. |
|  | **INDICATORS** | Annex to the IC contains proposed indicators for % of women aged 15-19 who are exposed to a FP message on any of the 8 media sources described, % of health workers trained on community sensitization (which includes adolescents), % schools with comprehensive CSE, and % facilities with youth corners |
|  | **INVESTMENT** | The IC notes that the National Health Financing strategy is currently under development. An Annex to the IC allocates a specific amount to this (USD252 236 over 3 years). Adolescents are also specifically included in action for collaboration to improve nutrition between the ministries of agriculture and education (USD72124 over 3 years). |
| **PAD Focus: Early Years growth and productivity project** | **CONTENT** | Adolescents are included in all sections of the document, including budget and M&E. They are included within a project subcomponent “Health facility interventions (USD1.6 million) to promote improved adolescent friendly health service package. The budget section notes that there is restricted fiscal apace and a host of competing priorities for investment in health and human development. Therefore, the project will support analytical work to guide community based SBCC targeting determinants of adolescent fertility in this project and multisectoral investments in support of the implementation of the National Strategy for adolescent girls and young women. Adolescents = 11-19 years. Includes “mobilizing boys and girls 11-19 to receive CSE to prevent early childbearing and improve uptake of SRH. |
|  | **INDICATORS** | Project beneficiaries including adolescent girls 11-19 who are beneficiaries of this project; adolescent girls who received iron-folate supplementation; adolescent girls aged15-19 who have begun childbearing. |
|  | **INVESTMENT** | Specifically included within a project subcomponent “Health facility interventions (USD1.6 million) to promote improved adolescent friendly health service package |
| **12. SIERRA LEONE** | | |
| **IC Focus: RMNCAH** | **CONTENT** | Adolescent health is embedded consistently throughout the document as part of the RMNCAH acronym and within separate sections. Reduction of the adolescent birth rate as one of 5 key targets. Discusses a multisectoral plan for AH, including adolescent friendly services, piloting demand-side financing interventions to reduce adolescent barriers to accessing service, and partnering with adolescent peer groups for behaviour change. |
|  | **INDICATORS** | One indicator specific to AH: reduction of the adolescent birth rate from 125.1 to 74 per 1000 women aged 15-19 years by 2021 |
|  | **INVESTMENT** | 1% of the national RMNCAH budget is allocated to interventions that target adolescents, not including SRH or maternal interventions |
| **PAD** | Not available | |
| **13. MADAGASCAR** | | |
| **IC Focus: RMNCAH-N** | **CONTENT** | Adolescent health appears in all main sections of the IC, but it is generally addressed as part of RMNCAH-N. A subsection is specifically dedicated to adolescent health in the "situational analysis" section of the RMNCAH-N |
|  | **INDICATORS** | The IC has a result monitoring framework that includes 7 impact indicators and 55 outcome indicators, 3 of which are entirely devoted to adolescent girls: These are: i) modern contraceptive prevalence among adolescents (15-19), ii) the proportion of adolescents (women 15-19) who have been tested for STIs/HIV and have withdrawn their results, and iii) the fertility rate of adolescents (15-19). |
|  | **INVESTMENT** | The total cost of implementing the investment plan was estimated at MGA 1,842,479,716,585 (or US$ 511,799,921) over five years. In this budget, MGA 72 billion (or US$ 20 million, equivalent to 3,9%) has been allocated for adolescent health |
| **PAD** | Not available | |
| **14. MALI** | | |
| **IC Focus: RMNCAH-N** | **CONTENT** | Adolescent health is included throughout the IC, and in separate sections. Detailed analysis of the adolescent health situation has been conducted. Plans include multisectoral interventions on adolescent and youth reproductive health (AYRH), including awareness-raising, training of CHWs on AYRH, strengthening sex education, RH education and prevention of STIs/HIV/AIDS among adolescents. |
|  | **INDICATORS** | AH is included with RMNCAH indicators but there is only one adolescent specific indicator in the performance framework: Proportion of adolescents who have already begun their fertile life |
|  | **INVESTMENT** | Provision is made for services to improve the reproductive health of adolescents and youth. This is estimated at 8.77 billion CFA francs, or approximately 2% of the overall budget for the strategy |
| **PAD Focus: Accelerating progress towards UHC** | **CONTENT** | Adolescents form part of the RMNCAH acronym, and do not have a separate section. PAD states that Mali has the highest adolescent age-specific fertility rate globally and the PAD mentions activities to affect behaviour change around FGM and increased demand for youth health services without attaching specific indicators or financing. |
|  | **INDICATORS** | DLI: % of girls, aged 15- 19, who are currently using any method of contraception |
|  | **INVESTMENT** | Adolescents are mentioned within RMNCAH under a subcomponent to increase quantity and quality of services delivered to the population (Performance-based payments (US$52.0 million: SDR 18.6 million (US$26 million equivalent) IDA, US$3 million GFF TF, and EUR 20.1 million (US$23.0 million equivalent) |
| **15. GUINEA** | | |
| **FIRST IC Focus: RMNCAH** | **CONTENT** | In its "Priorities" (section IV), the IC states that adolescent health will not benefit from a specific service package or budget, equating adolescent health needs with those of other targets (mother, child, newborn) |
|  | **INDICATORS** | Adolescents are not included |
|  | **INVESTMENT** | Adolescents are not mentioned in funding |
| **SECOND IC**  **[2015-2024]**  **Focus: RMNCAH** | **CONTENT** | A separate section is devoted to Adolescent/Youth Health. Adolescent health has been selected as one of the areas to be covered by high-impact interventions. The IC theory of change presents the reduction of adolescent fertility as a major impact indicator |
|  | **INDICATORS** | Proportion of adolescents aged 15-19 who have already begun their reproductive lives (page 39).  Prevalence rate of modern contraception among 15-19 year-olds. The objective is to reach a rate of 19.4% by the end of the IC implementation period (page 68).  Operational capacity of facilities offering adolescent health services. The target is to strengthen the capacity of 80% facilities |
|  | **INVESTMENT** | No particular investment is attached for adolescents |
| **PAD Focus: Health service & capacity strengthening project** | **CONTENT** | AH is mentioned briefly: The PAD notes that the country has a very high adolescent fertility rate. No further detail is given within document content. |
|  | **INDICATORS** | Adolescents are not included |
|  | **INVESTMENT** | Adolescents are not mentioned in funding |
| **16. CENTRAL AFRICAN REPUBLIC** | | |
| **IC Focus: RMNCAH** | **CONTENT** | Adolescent health is discussed extensively throughout the IC, sometimes in conjunction with maternal, neonatal, and child health, and sometimes separately with some subsections specifically dedicated to AH |
|  | **INDICATORS** | Specific indicators for AH include adolescent fertility rate, adolescent contraceptive use, adolescent HIV testing, post-abortion care, availability of adolescent health services (FP, HIV) |
|  | **INVESTMENT** | There is a specific budget for adolescent and youth health, representing 2,3% (about 3,463,321 USD / 150,621,801 USD) of the total budget. |
| **PAD Focus: Health system support & strengthening** | **CONTENT** | AH is mentioned briefly (GBV subcomponent includes raising awareness on GBV for adolescents - no specific indicators or funding attached, not included throughout the document) |
|  | **INDICATORS** | Within the PAD's M&E plan, “number of women and adolescents who received family planning services” is used as an indicator of service delivery. |
|  | **INVESTMENT** | Adolescents are not mentioned in funding |
